# The Vitamin D Receptor (VDR) Gene Polymorphisms in Turkish Brain Cancer Patients

**DOI:** 10.1155/2013/295791

**Published:** 2013-04-17

**Authors:** Bahar Toptaş, Ali Metin Kafadar, Canan Cacina, Saime Turan, Leman Melis Yurdum, Nihal Yiğitbaşı, Muhammed Oğuz Gökçe, Ümit Zeybek, Ilhan Yaylım

**Affiliations:** ^1^Department of Molecular Medicine, The Institute of Experimental Medicine, İstanbul University, Vakıf Gureba Caddesi, Capa, 34390 Istanbul, Turkey; ^2^Department of Neurosurgery, Cerrahpaşa Faculty of Medicine, İstanbul University, Istanbul, Turkey

## Abstract

*Objective*. It has been stated that brain cancers are an increasingly serious issue in many parts of the world. The aim of our study was to determine a possible relationship between Vitamin D receptor (VDR) gene polymorphisms and the risk of glioma and meningioma. *Methods*. We investigated the VDR Taq-I and VDR Fok-I gene polymorphisms in 100 brain cancer patients (including 44 meningioma cases and 56 glioma cases) and 122 age-matched healthy control subjects. This study was performed by polymerase chain reaction-based restriction fragment length polymorphism (RF LP). *Results*. VDR Fok-I ff genotype was significantly increased in meningioma patients (15.9%) compared with controls (2.5%), and carriers of Fok-I ff genotype had a 6.47-fold increased risk for meningioma cases. There was no significant difference between patients and controls for VDR Taq-I genotypes and alleles. *Conclusions*. We suggest that VDR Fok-I genotypes might affect the development of meningioma.

## 1. Introduction

The term of brain cancer is a heterogeneous group of neoplasms which occurs in intracranial tissue and the meninges. The incidence of malignant brain tumors is rare and approximately 2% of all cancers in adults [1, 2]. According to the result of several studies, the frequencies of brain tumors are of different rates according to countries worldwide. In addition, these differences can be observed among different ethnic groups in the same country [1]. Primary brain tumors are classified according to histopathological features like various other cancer types into the following major histologic groups: tumors of neuroepithelial tissue including astrocytoma, glioblastoma, oligodendroglioma, and ependymoma, tumors of meninges including meningioma and hemangioblastoma, and tumors of cellular region including pituitary tumors and craniopharyngioma [3]. Glioblastoma and meningioma are two of the most common and most malignant primary brain tumors. Gliomas are responsible for approximately 80% and meningioma is responsible for nearly 13–26% of all primary malignant brain tumors [4–6]. Primary brain tumors are a very heterogeneous group of diseases that are still not fully understood in their pathological mechanism. Despite all these developments, the contribution of genetic factors in the development of brain tumors is still not fully understood. Understanding of the genetic risk factors is very important for choosing the most suitable cancer treatment [6, 7].

Vitamin D is a steroid hormone that regulates several endocrine functions and cell functions such as, proliferation and differentiation. [8, 9]. The 1,25(OH)_2_D is one of potent regulators of cell growth and differentiation, which is an effect on cell death, tumor invasion, and angiogenesis, which makes it a candidate compound for cancer regulation [9]. It is known that vitamin D inhibits malignant cell proliferation and differentiation in several tissues such as breast, colon, skin, and brain [10]. The nuclear functions of vitamin D require binding to the vitamin D receptor (VDR) [11]. The vitamin D receptor (VDR) is involved in multiple pathways such as insulin-like growth factor (IGF) signaling; it also has a role in the inflammation and estrogen-related pathways that may be related to the prognosis of cancer [12, 13]. The gene encoding the VDR is located on the chromosome 12q12-14 [14]. Various studies suggested the effect of VDR gene polymorphisms in the development of several types of carcinoma such as breast, prostate, and colon carcinoma [9, 15–17]. One of the VDR gene polymorphisms is the Fok-I (rs2228570) polymorphism, which is located in exon 2 and results in an alternative transcription initiation site, leading to alter the activity of VDR protein. The polymorphic Fok-I site in exon 2 results in different translation initiation region due to thymine (T) to cytosine (C) substitution. The one of the most well known of these polymorphisms such as Taq-I (rs731236), which are located in exons 9, and a T/C nucleotide substitution (ATT to ATC) leading to a synonymous change at codon 352 (isoleucine) [18]. It has been reported that VDRs localized in neuronal and glial cells affect the metabolism of brain cells and change the expression of VDR [18, 19]. It has been reported that the potential effect of vitamin D on the treatment of cancer was first identified in myeloid leukemic cells [20]. Synthetic vitamin D analogues are among the preferred options in the treatment of central nervous system tumors. In addition, phase II clinical studies have correlated with positive effects of vitamin D therapy on glioblastoma cells [21–26]. The increased vitamin D synthesis in glioma cells after treatment can regulate cell proliferation [19]. However, the consist of this effect is required in VDR gene expression [20, 25]. Several studies have determined the contribution of VDR polymorphisms in various types of cancer. The aim of this study was to evaluate the association between polymorphisms in the VDR Fok-I and Taq-I and the risk of brain cancer in Turkish patients.

## 2. Materials and Methods

*Subject Selection.* We investigated the VDR Taq-I and VDR Fok-I gene polymorphisms in 100 brain cancer patients (including 44 meningioma cases and 56 glioma cases) and 122 age-matched healthy control subjects who were in the follow-up Cerrahpaşa Faculty of Medicine: Department of Neurosurgery in Istanbul University. The mean ages of glioma and meningioma patients and control group were 44.75 ± 15.63, 50.26 ± 12.68, and 47.22 ± 10.63 years, respectively.

The specimens were taken after obtaining informed consent, and the study was conducted prospectively. The Medical Ethics Committee of Istanbul Medical Faculty approval was obtained for the study. The protocol followed was consistent with the World Medical Association Declaration of Helsinki (Ethical Principles for Medical Research Involving Human Subjects).

### 2.1. Polymorphism Analysis

Blood specimens were collected in tubes containing EDTA, and DNA samples were extracted from the whole blood by a salting out procedure (Miller et al., 1988) [27]. Genotyping was performed by the polymerase chain reaction (PCR) and restriction fragment length polymorphism (RF LP). For Taq-I polymorphism, the following primers were used to amplify the VDR gene: 5′-CAG AGC ATG GAC AGG GAG CAA G-3′; 5′-GCA ACT CCT CAT GGG CTG AGG TCT CA-3′. For detection of the Taq-I RFLP, 50–100 ng genomic DNA was amplified with 1x polymerase chain reaction (PCR) buffer, 3 mM MgCl_2_, 0.2 mM of each dNTP, 0.2 mM of each primer, and Taq polymerase in a 50 *μ*L reaction volume. The PCR conditions were as follows: Initial denaturation step of 94°C for 4 min followed by 5 cycles of 94°C for 45 sec, 64°C for 60 sec, and 72°C for 2 min, and a further 25 cycles of 94°C for 30 sec, 64°C for 30 sec, and 72°C for 45 sec. PCR products were digested with *TaqI* restriction enzyme at 65°C, electrophoresed on 2% agarose gels, and stained with ethidium bromide. Genotypes were determined as TT (490, 245 bp), Tt (490, 290, 245, and 205 bp), or tt (290, 245, and 205 bp) for Taq-I polymorphism [28]. The primers (MBI Fermentas, Lithuania) for Fok-I polymorphism were 5′-GAT GCC AGC TGG CCC TGG CAC TG-3′; 5′-ATG GAA ACA CCT TGC TTC TTC TCC CTC-3′. The DNA template was amplified by PCR using 3 mM MgCl_2_, 0.2 mM of each dNTP, 0.25 mM of each primer, and Taq polymerase (MBI Fermentas, Lithuania) in a 50 *μ*L final volume. The PCR conditions involved an initial denaturation of 4 min at 94°C and followed by 30 cycles of 94°C for 1 min, annealing at 60°C for 1 min, and extension at 72°C for 1 min. A final extension step at 72°C for 4 min was also studied. PCR products were digested with *FokI *restriction enzyme (MBI Fermentas, Lithuania) at 37°C for 3 h followed by electrophoresis in a 2% agarose gel. The FF genotype (homozygote of common allele) shows only one band of 272 bp in agarose gel. The ff genotype (homozygote of infrequent allele) generates two fragments of 198 and 74 bp. The heterozygote displays three fragments (272, 198, and 74 bp) [29].

### 2.2. Statistical Analysis

Statistical analyses were performed using the SPSS software package (revision 16 SPSS Inc., Chicago, IL, USA). Data are expressed as means + SD. Differences in the distribution of genotypes or alleles between cases and controls were tested using the chi-square statistic. Odds ratios (ORs) and 95% confidence intervals (95% CI) were calculated to estimate the risk of glioma and meningioma. Values of *P* < 0.05 were considered statistically significant. Haplotype frequencies *D*′ and *r* ^2^ were calculated using Haploview 4.0 programme.

## 3. Results

The analysis included 100 brain cancer (including 44 meningioma cases, 56 glioma cases, and 122 healthy controls.  Table 1 shows the clinical characteristics of the study groups. Genotype and allele frequencies of meningioma cases, glioma cases, and controls are shown in Table 2. The risks of meningioma and glioma associated with VDR genotypes are shown in Table 3. There was a significant difference in the distribution of VDR Fok-I genotypes in meningioma patients but no significant difference, was detected in glioma patients (*P* > 0.05) (Table 2). VDR Fok-I ff genotype was significantly increased in meningioma patients (15.9%) compared with controls (2.5%), and carriers of Fok-I ff genotype had an increased risk for meningioma cases (*P* = 0.004) (*χ* ^2^: 10.33 OR: 6.470, %95 CI: 1.749–23.926). The VDR Taq-I genotype frequencies for meningioma, glioma, and control cases were not significantly different (*P* > 0.05). In addition, vitamin D haplotypes were evaluated for association with brain cancers. Haplotype analysis revealed that there was no relationship between VDR polymorphisms and brain cancers (for glioma cases: *D*′: 0.17, LOD: 1.08, and *r*-squared: 0.029 and for meningioma cases *D*′: 0.199, LOD: 1.06, and *r*-squared 0.034) (Tables 4 and 5).

## 4. Discussion

For the first time, we demonstrated the positive association of VDR Fok-I gene variants with meningioma cases. We also determined that VDR Fok-I ff genotype might affect development of meningioma, but we found that there was no statistically significant difference between VDR polymorphisms with glioma.

Vitamin D is an important factor in the regulation of cell division and differentiation. The VDR gene is a member of nuclear receptor superfamily. The VDR gene located on chromosome 12. For a long time, several polymorphisms in VDR gene have been investigated for functional significance and potential effects on disease susceptibility [17]. Many studies reported that vitamin D has an antiproliferative effect on many cancer types, which is promoting apoptosis in a variety of malignant cells, such as glioma, neuroblastoma, leukemia, lymphoma cells, breast cancer, and colon cancer [28, 30, 31], and numerous vitamin D analogs have been produced for the treatment of several cancer types. Alternations of vitamin D levels may be related to the changing in the expression of several transcription factors, cell cycle arrested proteins, growth factor, and other genes [32]. Several studies implicated that the ff and Ff genotypes of the VDR gene are associated with a decreased transcriptional activity. VDR Fok-I polymorphism changes the size of the VDR protein [33–36]. The shorter VDR variant could be less active; therefore, this variation may lead to more aggressive disease prognosis [28, 37]. There are some studies showing that vitamin D deficiency affected the brain morphology and cellular proliferation and growth factor signaling in other tissue [19, 38–40]. Brain tumors are relatively rare than other cancer types, but some of the brain cancers are fatal cancer types [41]. Meningiomas are one of the most frequent neoplasms of the brain tumors which account for nearly 13–26% and originate from the arachnoid cells or meningothelial cells [5, 42, 43]. Most of the meningioma cases are sporadic tumors. Today, we have a very little knowledge about genetic risk factors of meningioma. The pathophysiology of meningioma may be associated with a few genes, such as NF2, ATM, GST, CYP450, TP53, KRAS, and MNI. Especially, these genes are related to DNA repair, cell cycle regulation, tumor suppressor, and hormone metabolic pathways [44]. Malmer et al. reported that there may be a relationship between the ATM gene variants which regulate for cellular response against DNA damage and meningioma risk, [45]. Ting et al. reported that the activation of the ATM gene is mediated with VDR phosphorylation when genotoxic stress; furthermore, VDR gene mutation associated with inhibition of ATM gene expression [46].

Sadetzki et al. reported that the genotype of KRAS was related to the increased the risk of (nearly 2 fold) meningioma [47]. VDR expression rates were associated with KRAS mutation in several cancer types. Several studies suggest that cellular effects of VDR may be associated with MAPK signaling pathways, especially KRAS mutation in several cancer types such as breast and colorectal cancers [48, 49]. Some meningiomas might originate from arachnoids cells within the cranium, and other meningioma types may associated with increased bone density [50]. Furthermore, vitamin D also acts directly on osteoblasts, which is modulate differentiation, and regulates mineralization of the extracellular matrix in osteoblastic cells [33]. Previous studies reported that MN1 gene was a candidate genetic risk factor for sporadic meningioma cases [51]. Sutton et al. demonstrated that protein levels of MN1 was significantly in a relationship with vitamin D mediated transcription mechanism in osteoblastic cells [32]. All of these findings gave us an idea that vitamin D gene variations may be a genetic risk factor for the development of meningioma cases. Finally, we found that there was statistically significant difference between the control and meningioma patients for Fok-I ff genotypes. The individuals who had VDR Fok-I ff genotype had an increased risk for meningioma.

Despite an extensive literature study, there was no study that reported the relationship between VDR polymorphisms and brain cancer susceptibility. In conclusion, the present results suggest that the Fok-I polymorphism in the VDR gene may be related to the risk of meningioma. However, further studies with large number of subjects are needed to explain this kind of relationship between VDR-Fok-I genetic variants and meningioma risk.

## Figures and Tables

**Table 1:** General demographic informations and parameters of patients and control groups.

Parameters	Meningioma cases (*n* = 44)	Glioma (*n* = 56)	Controls (*n* = 122)
Age (years) (mean ± SD)	50.26 ± 12.68	44.75 ± 15.63	47.22 ± 10.63
Gender *n* (%)			
Male	18 (40.9%)	32 (57.1%)	51 (41.8%)
Female	26 (59.1%)	24 (42.9%)	71 (58.2%)
Histological characteristic of tumors			
Astrocytoma *n* (%)		11 (19.6%)	
Glioblastoma multiforme *n* (%)		22 (39.3%)	
Oligodendroglioma *n* (%)		8 (14.3%)	
Oligoastrocytomas *n* (%)		6 (10.7%)	
*Others *n* (%)		9 (16.1%)	

Values as average ± standard deviation. *Ependymoma, hemangioblastoma, paraganglioma, and so forth.

**Table 2:** Genotype and allele frequencies of meningioma cases, glioma cases, and controls.

SNP	Controls *n* (%) (*n* = 122)	Meningioma *n* (%) (*n* = 44)	*χ* ^2^	*P* value	Glioma *n* (%) (*n* = 56)	*χ* ^2^	*P* value
*Fok-I genotype							
FF	56 (45.9%)	19 (43.2%)			28 (50.0%)		
Ff	63 (51.6%)	18 (40.9%)	10.527	0.005	23 (41.1%)	4.598	0.1
ff	3 (2.5%)	7 (15.9%)			5 (8.9%)		
*Fok-1 alleles							
F	175 (71.7%)	56 (63.6%)			79 (70.5%)		
f	69 (28.3%)	32 (36.4%)	1.997	0.157	33 (29.5%)	0.052	0.818
^ #^Taq-I genotype							
TT	65 (53.3%)	23 (52.3%)			32 (57.1%)		
Tt	44 (36.0%)	18 (40.9%)	0.703	0.704	18 (32.2%)	0.275	0.872
tt	13 (10.7%)	3 (6.8%)			6 (10.7%)		
^ #^Taq-I alleles							
T	174 (71.3%)	64 (72.7%)			82 (73.2%)		
t	70 (28.7%)	24 (27.3%)	0.064	0.800	30 (26.8%)	0.137	0.710

Chi-square test was used to compare alleles and clinic pathological characteristics in the study group. *n*: number of individuals.

*For Fok 1 polymorphism (rs2228570): F is referred to as T allele, and f is referred to as C allele.

^#^For Taq 1 polymorphism (rs731236): T is referred to as T allele, and t is referred to as C allele.

Due to the same base changes T-C or C-T for both polymorphisms, it should be shown as the initial letter of the polymorphism.

**Table 3:** The risk of meningioma and glioma associated with VDR genotypes.

SNP	Controls	Meningioma	OR (95% CI)	*P* value	Glioma	OR (95% CI)	*P* value
Fok-1							
FF	56 (45.9%)	19 (43.2%)	1.050 (0.774–1.425)	0.756	28 (50%)	1.089 (0.787–1.508)	0.611
Ff + ff	66 (54.1%)	25 (56.8%)			28 (50)		
FF + Ff	119 (97.5%)	37 (84.1%)	6.470 (1.749–23.926)	0.001	51 (91.1%)	3.631 (0.899–14.665)	0.053
ff	3 (2.5%)	7 (15.9%)			5 (8.9%)		
Taq-I							
TT	65 (53.3%)	23 (52.3%)	1.022 (0.711–1.468)	0.909	32 (57.1%)	1.073 (0.810–1.421)	0.631
Tt + tt	57 (46.7%)	2 (47.7%)			24 (42.9%)		
TT + Tt	109 (89.3%)	41 (93.2%)	1.043 (0.943–1.153)	0.460	50 (89.3%)	1.005 (0.403–2.508)	0.991
tt	13 (10.7%)	3 (6.8%)			6 (10.7%)		

**Table 4:** Haplotype frequencies of glioma cases and controls.

Block	Haplotype	Case, control ratio counts	*χ* ^2^	*P*
Block 1				
TT	0.547	0.534, 0.553	0.114	0.7359
TC	0.170	0.198, 0.157	0.892	0.3448
CT	0.168	0.171, 0.166	0.017	0.8968
CC	0.115	0.097, 0.124	0.546	0.46

*For Fok-1 polymorphism (rs2228570): F is referred to as T allele, and f is referred to as C allele.

^#^For Taq-1 polymorphism (rs731236): T is referred to as T allele, and t is referred to as C allele.

Due to the same base changes T-C or C-T for both polymorphisms, it should be shown as the initial letter of the polymorphism.

**Table 5:** Haplotype frequencies of meningioma cases and controls.

Block	Haplotype	Case, control ratio counts	χ^2^	P
Block 1				
CT	0.543	0.512, 0.554	0.476	0.4904
CC	0.183	0.250, 0.159	3.581	0.0584
TT	0.153	0.125, 0.163	0.726	0.394
TC	0.121	0.114, 0.124	0.062	0.8032

*For Fok-1 polymorphism (rs2228570): F is referred to as T allele, and f is referred to as C allele.

^#^For Taq-1 polymorphism (rs731236): T is referred to as T allele, and t is referred to as C allele.

Due to the same base changes T-C or C-T for both polymorphisms, it should be shown as the initial letter of the polymorphism.
